# The influence of dilute aluminum and molybdenum on stacking fault and twin formation in FeNiCoCr-based high entropy alloys based on density functional theory

**DOI:** 10.1038/s41598-019-47223-3

**Published:** 2019-07-29

**Authors:** Peijun Yu, Yu Zhuang, Jyh-Pin Chou, Jie Wei, Yu-Chieh Lo, Alice Hu

**Affiliations:** 1 Department of Mechanical Engineering, City University of Hong Kong, Kowloon Tong, Hong Kong; 2 Department of Materials Science and Engineering, City University of Hong Kong, Kowloon Tong, Hong Kong; 3 City University of Hong Kong Shenzhen Research Institute, Shenzhen, P.R. China; 4 Department of Materials Science and Engineering, National Chiao Tung University, Hsinchu, 30010 Taiwan

**Keywords:** Metals and alloys, Atomistic models

## Abstract

Stacking faults, as defects of disordered crystallographic planes, are one of the most important slipping mechanisms in the commonly seen lattice, face-centered cubic (FCC). Such defects can initiate twinning which strengthens mechanical properties, e.g. twinning-induced plasticity (TWIP), of high entropy alloys (HEAs) at cryogenic temperatures. In this work, by using density functional theory (DFT), the twinning initiated from stacking faults is discussed with regard to two different solute elements, Al and Mo, in the FeNiCoCr HEAs. Our results show that adding aluminum (Al) has noticeable enhancement of twinnability while molybdenum (Mo) only induces more stacking faults in the FeNiCoCr-based HEAs.

## Introduction

High entropy alloys (HEAs), a new class of metallic materials constructed by Yeh *et al*.^1^, composed with five or more elements, equi- or near equi-atomic percentage, represents numerous novel properties for structure materials including defeating the trade-off between strength and ductility^2–5^. Other than the well-studied defects, such as dislocations, in many materials, some of the FCC HEAs appear to conserve nano-twins with stacking faults while some of them tend to contain stacking faults only. These phenomena were reported in deformation-induced nano-twins in HEAs resulting in significant enhancement of ductility due to twinning-induced plasticity. The strength of such HEAs are also enhanced because of the interactions between twin boundaries and dislocations in FeCrNiCoMn^4,6^, FeCrNiCo^7^ and CrCoNi^8,9^. Alloys with low stacking fault energies could deform with deformation twins and enhancement of strength without sacrificing ductility tremendously^10^. Annealing and deformation twins were both found in Al *x*FeNiCoCr in the experiments conducted by Liaw *et al*.^11^ when the mole fraction *x* of Al is constrained under 0.45 to get single-phase FCC strucutres. However, in the study of MnFeNiCoCr completed by Mao *et al*.^12^, stacking faults are found in the deformed samples, the stacking faults were reported to dominate the plasticity in the initial deformation. In Niu *et al*.’s study of CrCoNi alloys^13^, they found a distinct lower-energy HCP phase. In Patriarca *et al*.’s work of FeNiCoCrMn^14^, they not only showed the low stacking fault energies using DFT calculations, but also indicated that the presence of Co atoms are favored near the stacking faults, which could reduce the intrinsic stacking fault energy by almost 55%. However, the effects of solute components on the twin and stacking fault formation in HEAs are less discussed currently. Therefore, systematic investigation of the stacking fault formation induced by solute atoms would be a support to the understanding of twinning and phase transition in FeNiCoCr-based HEAs.

The intrinsic stacking fault energy (ISFE), extrinsic stacking fault energy (ESFE) and HCP phase transition energy (PTE) are calculated in the current work using the first-principles method in the model constructed via special quasi-random structure (SQS) technique^15,16^. At first, we evaluate the stability of intrinsic and extrinsic stacking faults and HCP phase transition in FCC Al *x*FeNiCoCr using ISFE, ESFE and PTE. The minimum energy paths are then calculated to quantify the ease of transitions between these states by slipping of close-packed atomic planes. Finally, we try to evaluate the effects of Al and Mo atoms on the tendency of forming twins and stacking faults in such HEAs, by the quantification of the lattice distortion in each structure using the mean-square atomic displacement (MSAD).

## Methods

Density function theory (DFT) is used to calculate the formation energies of various crystal structures in this study^17^. The plane-wave based Vienna *Ab initio* Simulation Package (VASP) is employed for the simulation with the projector augmented wave (PAW) method^17^ as the pseudo-potential treatment. The generalized gradient approximation (GGA) parameterized by Perdew, Burke and Ernzerhof (PBE)^18^ is chosen as the exchange-correlation function. Full valence electron of the composited elements (Fe, Cr, Ni, Co, Al/Mo) for these prototype alloys are considered and have valence electron number of 8, 6, 10, 9, 3/6, respectively^19^. Although usually the cutoff radius of plane-wave energy chosen as 350 eV, which is the maximum cutoff radius of Ni among considered components (269 eV) with a pre-factor of 1.3 is large enough for alloy studies, our test result in Supplementary Fig. 2 shows that if the cancellation effect is taken out of consideration, a plane-wave energy cutoff radius of 400 eV is necessary in order to eliminate the uncertainties. Monkhorst-Pack k-point spacing of 0.2 Å^−1^ is used in our study. The tolerance of the energy and force convergence were 1.0 × 10^−5^ and 1.0 × 10^−4^, respectively. In order to represent the chemical disorder of HEAs and solid solution state of Al and Mo in the FeNiCoCr matrix, the Alloy Theoretic Automated Toolkit (ATAT) is used to build the supercells containing 96 atoms (Fe_22_Ni_22_Co_22_Cr_22_X_8_, X stand for Al/Mo) with SQS^20,21^. Supercell models were used in this study^22^, the slab models are built with vacuum added in the 111 direction. The thickness of vacuum is tested and chosen as 26 Å so that the pseudo-interactions between the repeated slabs in the 111 direction due to the periodic boundary condition (PBC) can be eliminated. Detailed vacuum thickness tests and selection can be seen in the Supplementary Fig. 3.

The size of systems would also have effects on the calculation results. In Zhao *et al*.’s work^23^, they carried out the molecular dynamics (MD) simulations of NiFe solid solution alloys with various stacking fault areas. In their work, they presented the distributions of SFE in NiFe with stacking fault areas of 96.57, 536.52, 2146.08, 8584.32, 19314.72 and 34337.28 Å^2^. The standard deviation decreases rapidly with increasing stacking fault area. That means, especially for the multicomponent solid solution, larger size systems should be considered for eliminating the uncertainties of SFEs caused by local chemical environments. However, the number of atoms in DFT calculations are usually constrained at tens or hundreds. Therefore, instead of using systems as large as they used in the MD simulation, we conducted multiple samples to get the averaged values of SFEs and we believe the standard deviation would be smaller if very large model could be used in the DFT calculations.

**Figure 1:**
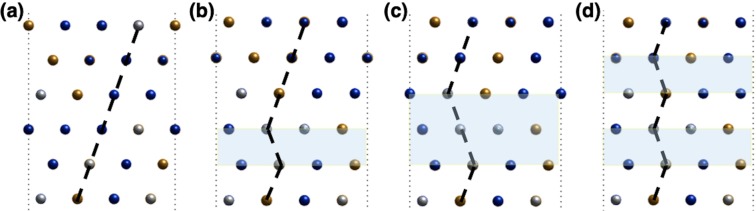
Schematic for lattice structures with slipped planes and planar symbols illustrated. (**a**) Pristine FCC, (**b**) FCC with intrinsic stacking fault (ISFE), (**c**) FCC with extrinsic stacking fault (ESFE), (**d**) HCP caused by two stacking faults introduced into FCC (PTE).

Figure 1 shows the stacking sequence of atomic planes in each structure and the atom mapping. The SQSs with FCC lattice were firstly generated, then the Shockley partial dislocations with Burger’s vector

1 $$\mathop{{b}_{p}}\limits^{\rightharpoonup }=(a/6)\langle {\rm{11}}\bar{2}\rangle $$

were assigned to the atoms in 3^rd^, 4^th^ and 5^th^ layers to create the desired structures.

## Results and Discussion

In Table 1, the lattice parameters of each structure were presented to determine the effect of stacking faults on the lattices. In general, adding Mo or Al atoms into FeNiCoCr will both increase the lattice constant. The Mo-containing HEAs would have larger lattice constant than the Al-containing ones because of the larger atomic radius of Mo. When introducing partial dislocations into the FCC structures, lattice parameters are slightly elevated due to the reduction of geometrical symmetry. Compared to the theoretical determination of FeNiCoCr lattice parameters in Zhang *et al*.’s work^23^, adding Al atoms slightly decreases the lattice parameters while adding Mo would have the opposite effect. Molybdenum, as one of the transition metals, has similar atomic and ionic radius to the Fe, Ni, Co, Cr atoms in the matrix. However, aluminum has similar atomic radius and much smaller ionic radius. The distinct ionic size of aluminum atoms could serve as one of the reasons of decreased lattice constant. On the other hand, Mo has larger atomic and ion radius than the matrix atoms. These could result in different ways of lattice distortion.

**Table 1:** Lattice parameters and the cohesive energies determined by DFT calculations for different structures.

Structures	FCC	Mo	ISF	Mo	ESF	Mo	HCP	Mo
	Al		Al		Al		Al	
Lattice parameter (Å)	3.4954	3.5104	3.4956	3.5145	3.4970	3.5152	2.4763 4.2461	2.4909 4.0838
Cohesive energy (eV/atom)	−4.5327	−4.6677	−4.5346	−4.6682	−4.5317	−4.6682	−4.5427	−4.6763

“Al” and “Mo” categorize the HEAs containing aluminum and molybdenum solutes in FeNiCoCr matrix.

The cohesive energies in Table 1 are calculated using

2 $${E}_{coh}=(\sum {E}_{atom}-{E}_{bulk})/N$$

where *E*_*atom*_ represents the energies of each species at single-atom states and *E*_*bulk*_ is the energies of each structure at its alloying state. The energy differences are averaged by *N* the total number of atoms in the model.

The stacking fault energies are calculated using

3 $${\gamma }_{isf}=({E}_{isf}-{E}_{0})/{A}_{0}$$

Where *E*_*isf*_ and *E*_0_ the total energies of faulted and pristine supercells, respectively, *A*_0_ is the area of stacking fault.

As shown in Table 2, the surprisingly negative stacking fault energies of both samples suggest that at cryogenic temperature, faulted FCC is more stable than pristine FCC for both Al- and Mo-containing HEAs. In Zhao *et al*.’s work^22^, the results of the axial interaction model (AIM) and the one dimensional axial next-nearest neighbor Ising (ANNNI) model both showed very low even negative stacking fault energies for the face-centered cubic HEAs. The results from their ANNNI models and our results indicate that HCP is more favorable than FCC energetically and the total energies for both samples experience rebounds when introducing ESFs into them. Apparently, the cohesive energies of Al-containing HEAs increase dramatically with ESF structures (go to positive value). The HCP phase turns out to be the universally lowest energy state of both HEAs. However, the DFT calculations in this study were carried out with cryogenic temperatures, which are not able to predict the phase stability of elevated temperatures. In the Supplementary Fig. 4, the temperature dependent phase stability of the studied HEAs is presented. It can be seen that with the temperature increases, the FCC phase becomes more stable than the HCP phase. Notably, for all the calculated structures (ISF, ESF and HCP), the Al-containing ones always cause greater changes in cohesive energies. These evidences all could point to the fact that the phase stability of FeNiCoCr-based HEAs would be decreased by adding Al atoms.

**Table 2:** Stacking fault energies and energy differences between FCC and HCP structures showing the stabilities of each deformed structures.

Structure	γ_isf_		γ_esf_		E_hcp_	
	Al	Mo	Al	Mo	Al	Mo
SFE (*meV*/Å)	−2.2	−0.56	1.1	−0.62	−12	−9.6
SFE (*mJ*/*m*^2^)	−35	−9	18	−10	−185	−153

**Figure 2:**
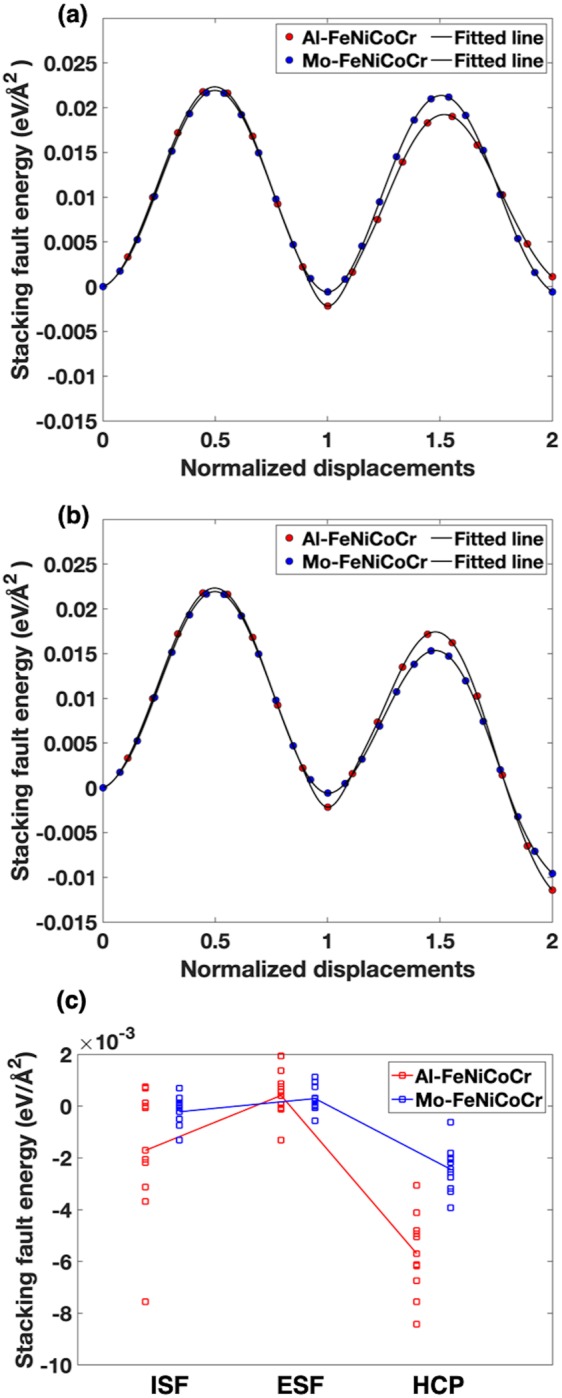
Calculated minimum energy paths (MEPs) for transition states between each state. The red and blue dots represent the calculated energy points. (**a**) Energy barriers from FCC to ISF to ESF, red and blue curves represent the aluminum and molybdenum composited HEAs, respectively. (**b**) Energy barriers from FCC to ISF to HCP, red and blue curves represent the aluminum and molybdenum composited HEAs, respectively.

In order to examine the statistical uncertainties caused by the local chemical environments, the SFEs by the slipping of different atomic layers were calculated. As shown in Table 3, the stacking fault energies at different fault layers were carried out. The averaged SFEs for Al_0.36_FeNiCoCr and Mo_0.36_FeNiCoCr are −2.46 *meV*/Å^2^ and −0.28 *meV*/Å^2^, respectively. The individual SFEs diverse greatly and the averaged value still show that the SFE of Al_0.36_FeNiCoCr is much lower. Local chemical environments would have significant effects on the results of stacking fault energies, especially in the Al-containing HEAs. Furthermore, in order to study the uncertainties caused by atomic configurations, 10 different SQSs were taken into consideration for each proposed structure. Figure 2(c) shows the averaged value for each structure. The mean values show a clear trend that Al_0.36_FeNiCoCr has lower energy of ISF, HCP when Mo0.36FeNiCoCr has lower energy of ESF. When compare these results to the work of others, e.g. CoCrNi and FeNiCoCr alloys^24–26^, it seems that aluminum tends to decrease the SFEs while molybdenum tends to increase the SFEs.

**Table 3:** Stacking fault energies calculated for different slipping layers in the 6-layer slab models for Al and Mo doped HEAs.

System	Al_0.36_FeNiCoCr					
Layer	1^st^	2^nd^	3^rd^	4^th^	5^th^	6^th^
γisf (meV/Å^2^)	−7.0	0.62	0.5	−2.2	−2.1	−4.6
γisf (mJ/m^2^)	−112	10	8	−35	−33	−74
**System**	**Mo**_**0.36**_ **FeNiCoCr**					
γisf (meV/Å^2^)	−1.9	−0.12	−0.37	−0.56	0.56	0.69
γisf (mJ/m^2^)	31	−2	−59	−9	9	11

In addition to the cohesive energy calculations, the minimum energy paths and the energy barriers during the shifting of atomic planes for each slipping mechanism were investigated. The activation energies are presented in Fig. 2(a,b). The nudged-band elastic (NEB) method is used in the energy barrier calculation. The combination of the climbing image method and the better tangent definition gives more accurate saddle points with fewer images and larger displacement intervals, compared to the originally compiled elastic band (EB) method in VASP^19,27–32^.

As shown in Fig. 2(a), the first saddle of creating ISF in Al_0.36_FeNiCoCr is around 0.023 eV/Å^2^. The Mo-containing samples have slightly lower barrier than the Al-containing samples. From the second saddles for ESFs, the energy barriers basically keep the same value as those for ISFs. Either the formation energies or the activation energies of Mo-containing HEAs have small changes from one structure to another. This validates what is stated in the previous discussion, the Al-containing HEAs are less stable than that the Mo-containing ones, as per se, adding Al enhances the meta-stability and propensity of forming stacking faults and nano-twins in the FeNiCoCr-based HEAs^32^. The second saddle in Fig. 2(b) shows great propensity of both samples of forming HCP phases in FCC through generating stacking faults although in experiments the HCP phases are not usually probed because the continuous deformation would eventually induce large amount of stacking faults or nano-twins.

**Figure 3:**
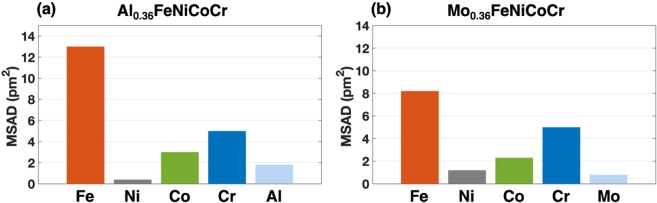
Mean square atomic displacement (MSAD) that shows the difference of lattice distortion between aluminum and molybdenum composited HEAs in perfect FCC structures. (**a**) and (**b**) represent the MSAD of each element after structure optimization of Al_0.36_FeNiCoCr and Mo_0.36_FeNiCoCr, respectively.

**Figure 4:**
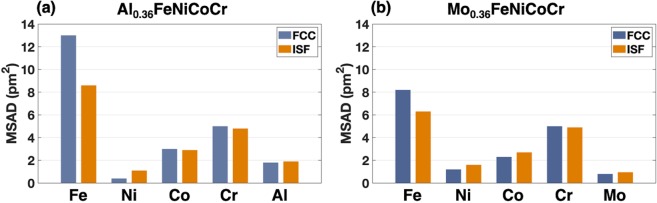
Comparison of MSAD of each element before and after intrinsic stacking fault is introduced for both aluminum and molybdenum composited sample. (**a**) and (**b**) represent the MSAD comparison of optimized FCC and ISF structures of Al_0.36_FeNiCoCr and Mo_0.36_FeNiCoCr, respectively. Black bars represent the FCC with optimized structures. Orange bars represent the ISF with optimized structures.

Lattice distortion could be the origin why Al atoms decrease the phase stability of FeNiCoCr-based HEAs. The mean square atomic displacement (MSAD) is extracted from the DFT calculations for each atom. The MSAD in this work was carried out using

4 $$MSAD={(x-{x}_{0})}^{2}=\frac{1}{N}\sum _{n=1}^{N}\,{({x}_{n}(t)-{x}_{n}(0))}^{2}$$

where the *x* and *x*_0_ represent the coordinates of atoms at the initial and terminal states, respectively. For the faulted configurations discussed in this work, the cell-shape optimized models with rigid displacements inserted into the optimized FCC structure were used as the initial states and then in the same supercells ionic positions optimization were carried out as the terminal states to ensure the coordinates in the MSAD determination are in the same space (supercell). From Fig. 3, it is obvious that all the atoms except nickel and chromium atoms have larger MSAD in the Al_0.36_FeNiCoCr than those in the Mo_0.36_FeNiCoCr. The lattice distortion induces higher atomic stresses which results in the higher energy barriers during stacking faults generation. In Okamoto *et al*.’s work on correlation between atomic displacement (AD) and solid solution strengthening (SSS). They proposed a proportional relation between MSAD^1/2^ and the yield strength of quaternary HEAs^33^. Our results show that stacking faults also have their influence on the local lattice distortion. In Fig. 4, the MSADs of each element are compared along with the difference between pristine FCC structures and ISF structures. It is clear that after the generation of ISF, the previous lattice distortion got decreased for a certain level, especially for Fe atoms, which could be one of the factors leading to negative stacking fault energy. In addition, the MSAD differences of iron atoms between FCC and ISF structures in the Al_0.36_FeNiCoCr is larger than which in the Mo_0.36_FeNiCoCr. As stated in the previous paragraph, the Al atoms would decrease the stability of HEAs and the changes in lattice distortion induced by stacking faults are also more dramatic compared to the Mo-containing HEAs. The decreased lattice distortion contributes to the lower stacking fault energy of the Al_0.36_FeNiCoCr. The 1^st^ nearest neighbors of the Al atoms of the faulted atomic planes are also checked to find out the reason iron atoms experience larger MSADs change before and after ISFs. In our SQS models of Al_0.36_FeNiCoCr and Mo_0.36_FeNiCoCr, Al and Mo atoms take substitution with each other in the same configuration. There are three pairs of Al/Mo-Fe nearest neighbors between the two faulted atomic planes (nearest neighbors in the same close-packed plane are not counted). While in the ISF structures, the Al/Mo-Fe neighbor numbers are reduced to one which reduces the atomic offset of iron atoms.

In Wen *et al*.’s publication of generalized stacking fault energy, they found that in the nickel based alloys, the atomic radius difference Δ*R* as well as valence electron difference Δ*VEC* between solute atoms and solvent atoms all have influence on stacking fault energy in FCC alloys^34^. In their work, larger Δ*R* result in lower stacking fault energy and larger Δ*VEC* also tend to drag down the stacking fault energy. As shown in Table 4, in our current study, when we treat solvent atoms valence electron count as the mean value of each element in the quaternary matrix alloy, the Δ*VEC* are 5.25 and 2.25 for Al_0.36_FeNiCoCr and Mo_0.36_FeNiCoCr, respectively. And the behaviors of stacking fault energies with respect to the Δ*VEC* agree with their proposition. The Δ*R* of the Al and Mo are 17.5 pm and 13.5 pm, respectively, are also consistent with their findings. For the species considered in this work, if geometry sizes of atoms are considered, we think the difference between the radius of ionic radius which are Δ*R*_*ionic*_ = −21 *pm and* −3 *pm* for Al and Mo, respectively, are also one of the factors that can affect the stacking fault energy of alloy systems. The smaller size of Al nuclei compared to Mo shows ease and tendency of forming stacking faults. In Ma *et al*.’s report^11^, they showed that when the mole fraction of Al in FeNiCoCr-based HEA is above 0.45, the material would tend to form BCC/B2 phase in it. That suggests that although Al itself is naturally FCC metal, adding Al to an FCC alloy matrix will induce BCC/B2 phase separation, says, adding Al will stabilize the BCC structure in FCC HEAs. In King *et al.’*s work of Al_*x*_FeNiCoCr alloys at atomic scale, they give a detailed study of Al content effects on the phase stability of HEAs^35^. In their work, the HEAs tend to remain disordered FCC when the Al content stays below 0.5. And they predict a transition between and FCC-based structure to the partially ordered BCC structure with increasing Al content which has great agreement with the experiments. They also investigated the ordering of the FeNiCoCr FCC phase in a Cu3Au (L1_2_)-type structure which is devoid of Al. Note that if take the partially ordered structures in the FCC HEAs, the results of SFEs of our systems would differ^36^. However, we here use a lower concentration of Al contents and discuss the influence of Al and Mo atoms in a completely random solid solution.

**Table 4:** The stacking fault energy (SFE) and valence electron counts for various HEAs. SFEs of quaternary HEAs calculations are conducted by Y.H. Zhang *et al*. in the literature (22).

Systems	Composition near ISF	γisf (mJ/m^2^)	γisf (meV/Å^2^)	Valence electron count (VEC)
FeCrNiCo (22)	Fe8Co6Cr8Ni10	−117	−7.3	8.31
	Fe8Co8Cr8Ni8	−82	−5.1	8.25
	Fe9Co10Cr7Ni6	−180	−11.2	8.25
Al_0.36_FeCrNiCo	Fe6Co9Cr4Ni9Al4	−35	−2.2	7.97
	Fe5Co6Cr6Ni11Al4	−55	−3.4	7.88
Mo_0.36_FeCrNiCo	Fe6Co9Cr4Ni9Mo4	−9	−0.56	8.34

In addition, molybdenum, as a naturally BCC metal, could also stabilize the BCC structure in FCC HEAs. We think that one of the reasons for the tendency of forming twins or stacking faults caused by Al or Mo is the ratio of ionic radii to van der waals radii, *k* = 0.350 *and* 0.489 for Al and Mo, respectively.

## Conclusion

In this work, the ISFEs, ESFEs and PTEs and their activation energies in single-phase FCC FeNiCoCr-based HEAs with solid solution components (Al/Mo) were calculated using DFT. The MSAD, ionic radius and VEC were used in our study to find the effects on stacking fault formation by adding different types of solute atoms into the quaternary HEA matrix. The temperature-dependent phase stability of FCC and HCP phase in this HEA was also calculated. The negative ISFE shows the great propensity of forming stacking faults in the HEAs used in our work. The even lower ISFE caused by Al solutes took responsibility for the high propensity of twinning in Al_0.36_FeNiCoCr which agrees with the experimental observation from the others’ work^37^. The higher ESFE (obvious in Al_0.36_FeNiCoCr while slight in Mo_0.36_FeNiCoCr) implies that forming twins from growth of stacking faults may not be the energetically favorable way for deformation. On the contrary, the surprisingly low PTE and energy barriers of both samples show strong evidence that parallel stacking faults which tends to form one after another by skipping one atomic plane is more likely to be the deformation path for twin growth. We also showed that the lattice distortion is released (especially Fe atoms) after introducing stacking faults which partially contributes to the negativity of SFE. From the aspect of atomic electron structures and geometry size of atoms, higher value of differences in nucleus radius and valence electron numbers between solute atoms and solvent atoms tends to form more stacking faults in FCC HEAs. To sum up, the solid solution HEAs with Al or Mo are both likely to form annealing and deformation stacking faults while Al_0.36_FeNiCoCr would reserve more stacking faults and nano-twins during annealing and form more nano-twins during deformation from the perspective of ionic to atomic radius ratio and valence electron count.

## Supplementary information

The influence of dilute aluminum and molybdenum on stacking fault and twin formation in FeNiCoCr-based high entropy alloys based on density functional theory

## Data Availability

The authors declare that the data supporting the findings of this study are available within the paper and supplement. Also, the data that support the plots within this paper are available from the corresponding author upon reasonable request.
