# Supplementary material for: The influence of dilute aluminum and molybdenum on stacking fault and twin formation in FeNiCoCr-based high entropy alloys based on density functional theory
Source: Sci Rep. 2019 Jul 29;9:10940. doi: 10.1038/s41598-019-47223-3 (PMC6662672; doi:10.1038/s41598-019-47223-3)
Supplement: Supplementary file 1 — The influence of dilute aluminum and molybdenum on stacking fault and twin formation in FeNiCoCr-based high entropy alloys based on density functional theory [file 41598_2019_47223_MOESM1_ESM.docx]

**The influence of dilute aluminum and molybdenum on stacking fault and twin formation in FeNiCoCr-based high entropy alloys based on density functional theory**

Peijun Yu ^a^, Yu Zhuang ^a^, Jyh-Pin Chou ^a^, Jie Wei ^a^, Yu-Chieh Lo ^d^*, Alice Hu ^a,b,c^*

^a^ Department of Mechanical Engineering, City University of Hong Kong, Hong Kong

^b^ Department of Materials Science and Engineering, City University of Hong Kong, Hong Kong

^c^ City University of Hong Kong Shenzhen Research Institute, Shenzhen, P. R. China

^d^ Department of Materials Science and Engineering, National Chiao Tung University, Taiwan

**Supplementary Information**

The theoretical study performed by Ma et al. ^1^ suggested that at equilibrium volume, the local magnetic moments almost vanish in the HEAs, with disordered local moment (DLM), ferromagnetic (FM) and anti-ferromagnetic (AFM) considered. In S.Zhao et al.’s work ^2^, the non-magnetic (NM) and AFM calculation of both FCC and HCP HEAs were carried out and their results show that the HCP structure is always energetically favorable for HEAs, which implies that the spin-polarization effect taken into account still leads to a lower stacking fault energy. Although in M.B. Kivy et al.’s work, they showed 12 atoms per layer with 9 layers models (108 atoms in total) for stacking fault energies, twinnability calculations of CoCrFeNi-based FCC HEAs based on DFT are valid ^3^, we still test the size effect of supercell.

In the supplementary information, we show the test of thicker model of each configuration. The Supplementary Fig. 1 shows the detailed configuration and cell shape, in order to examine the effect of model size, especially the thickness to see the effects from the free surfaces of the slab model, and the magnetic effect in formation energy of different faulted configurations. Supplementary Fig. 2(a) shows that the energy of these studied configurations have the same trend with the 96-atom models we used in this work, the formation energy of ISF is negative, ESF is positive and HCP has the lowest energy, though the local chemical order does effectively affect the stacking fault energy which drives the energy to a lower value. The vacuum layers added in the 96-atom and 192-atom models are 26 Å. From these test results we can assure that in the studied structures of this work, a 96-atom model is large enough and would not cause too heavy computational cost.

The choice of the vacuum thickness is also discussed here. The Al_0.36_FeNiCoCr HEA was used in the tests of various vacuum thickness. We used the same atomic distribution for all the vacuum tests in this study. As can be seen in the supplementary Figure 3, the vacuum thickness from 10 to 36 Å with iteration of 2 Å were tested. For the calculations with vacuum thinner than 18 Å, the cohesive energies of the samples possess drastic fluctuation which means the pseudo-interaction would affect the results of this study. Starting from vacuum thickness of 22 Å, the cohesive energies converge and fluctuate slightly. With these results, we can assure that the vacuum thickness of 26 Å in this study would provide reliable results.

Calculations based on the density functional perturbation theory (DFPT) were performed thusly with a $2\times2\times2$ k-point mesh for FCC and HCP supercells without vacuum layer added in the z-direction to determine the phonon density of state by determining the Hessian matrix. One should notice that unlike the NEB calculations, periodic boundary conditions (PBC) are used in the DFPT calculations and the phonon determinations are based on non-spin-polarized calculations. From the work of Niu et al.^4^, the cross-point of FCC and HCP temperature-dependent phase stability could be skewed due to the strong magnetism of Fe atoms. However, with the limited computational power currently, we are not able to reproduce the spin-polarized DFPT calculations in these quinary systems. The results from phonon calculations could still be considered as a qualitative determination of competing phase stability between FCC and HCP. Due to the lack of symmetry of the random distribution of each element, each atom is assigned 6 perturbation which correspond to $\pm x, \pm y,\pm z$. That says, for each structure at tilted volumes, 576 images calculation are conducted. With DFPT, the calculation based on phonon frequencies such as phonon density of states (PDOS), temperature-dependent free energy and thermal properties like heat capacity and thermal expansion could be determined. Phase stability calculation was conducted with the quasi-harmonic approximation (QHA) using Phonopy^5^, an open source package interfaced with VASP for phonon calculations at harmonic and quasi-harmonic levels^6–8^. The QHA calculations use 5 volume images with a lattice parameter ranging from 0.98 to 1.02 near the equilibrium state. Thermal properties at constant pressure could be obtained with derivation of volume-energy functions and Helmholtz free energies followed by fiiting pV terms in the formulism of Gibbs free energy^2,9^.

**
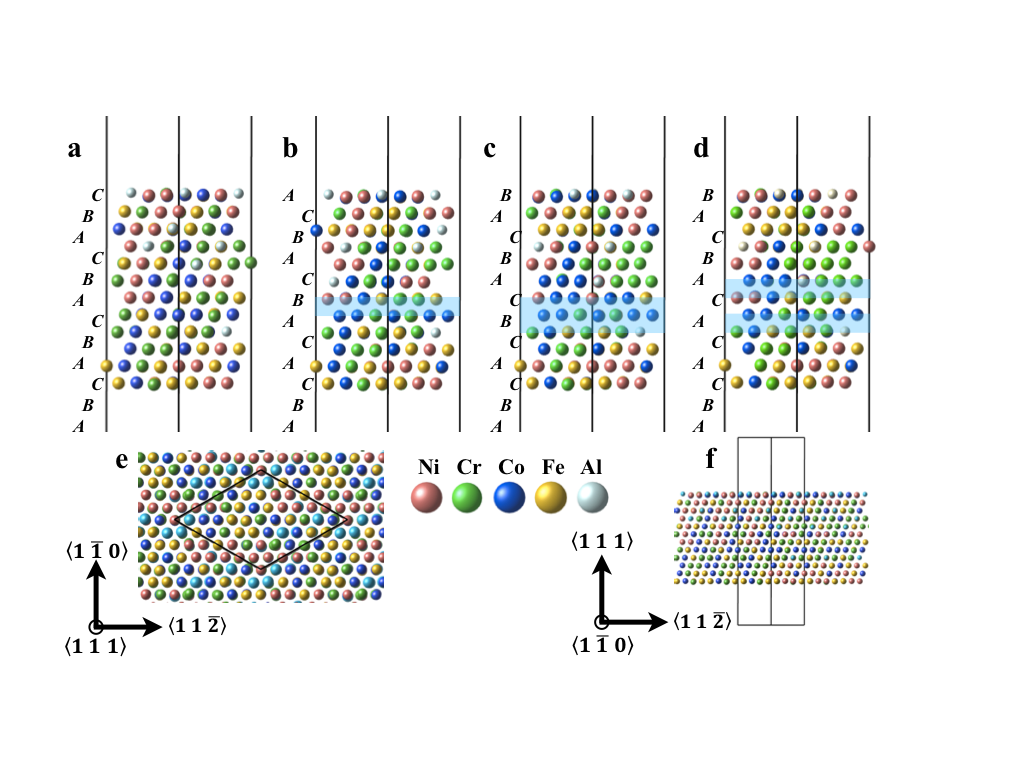
**

**Supplementary Figure 1. Configuration of the studied HEA structures with larger super-cell (192 atoms) with same concentration of each component.** (a-d) show the atomic configurations of the FCC, ISF, ESF and HCP, respectively. (e) shows the top view of the close-packed atomic plane and the selected cell. (f) shows the side view of the slab model and illustrates the thickness of vacuum layers in it.

**Supplementary Figure 2. Tested ground state energies of each state with convergence results of spin-polarized tests and maximum energy cutoff tests.** (a) shows the formation energy of predicted deformation paths of test larger super-cell with 192 atoms which still has the same trend of 96 atoms model. (b) and (c) show the choice of plane-wave energy cutoff radius and difference between number of electrons in up and down spin components in the spin-polarized convergence test.


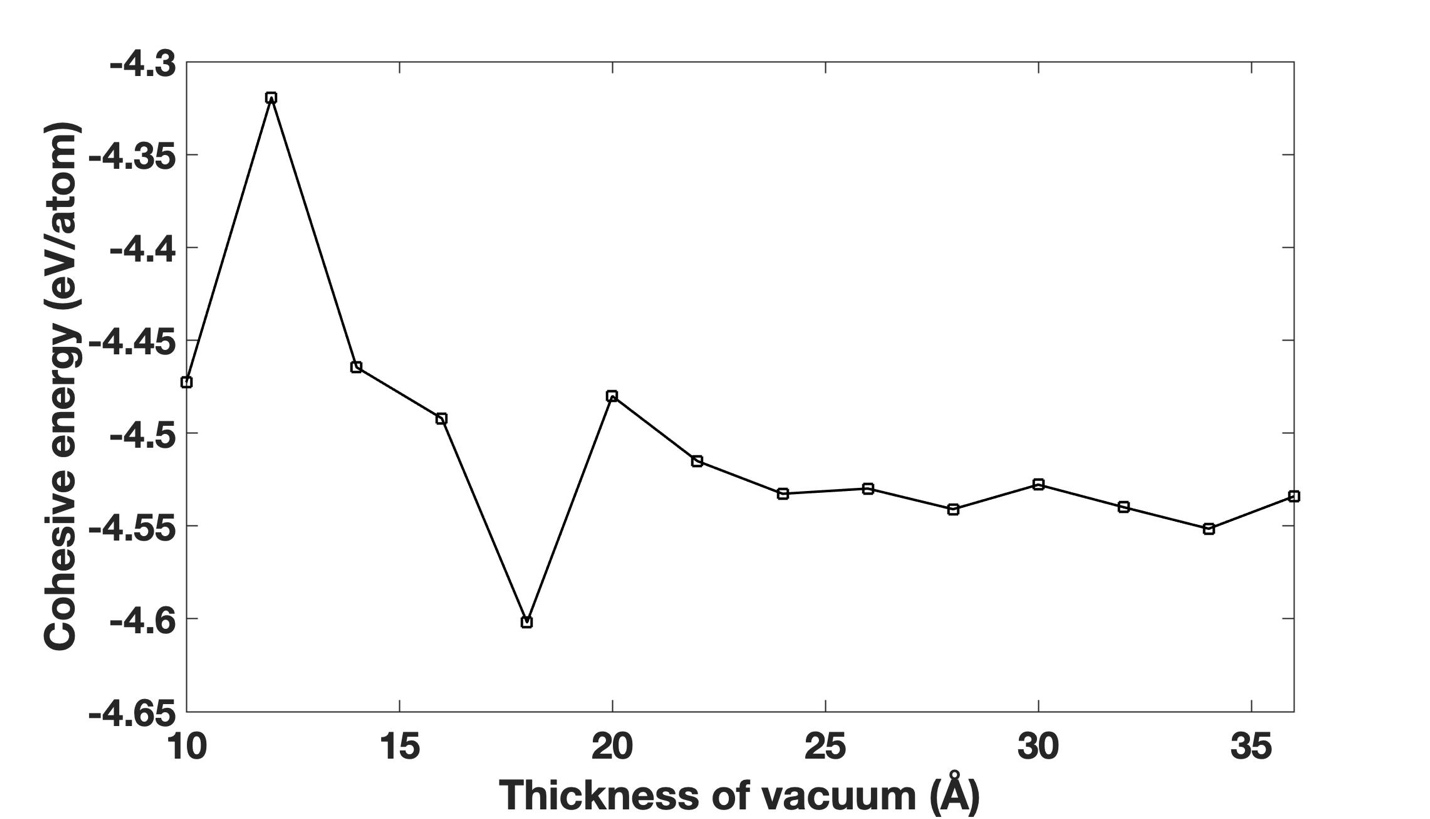


**Supplementary Figure 3. Test results of various thickness of vacuum in the 96-atom supercells.** The cohesive energies in the unit of eV/atom are presented with regard to the thickness of vacuum range from 10 to 36 Å. All the results were obtained from the DFT calculations of 96-atom Al_0.36_FeNiCoCr HEAs with same atomic distribution and different vacuum thickness.

**Supplementary Figure 4. Differences between temperature-dependent Gibbs free energies of FCC and HCP phases of FeNiCoCrAl_0.36_ high-entropy alloys.**

1. Ma, D., Grabowski, B., Körmann, F., Neugebauer, J. & Raabe, D. Ab initio thermodynamics of the CoCrFeMnNi high entropy alloy: Importance of entropy contributions beyond the configurational one. *Acta Mater.* **100**, 90–97 (2015).

2. Zhao, S., Stocks, G. M. & Zhang, Y. Stacking fault energies of face-centered cubic concentrated solid solution alloys. *Acta Mater.* **134**, 334–345 (2017).

3. Beyramali Kivy, M. & Asle Zaeem, M. Generalized stacking fault energies, ductilities, and twinnabilities of CoCrFeNi-based face-centered cubic high entropy alloys. *Scr. Mater.* **139**, 83–86 (2017).

4. Niu, C., Larosa, C. R., Miao, J., Mills, M. J. & Ghazisaeidi, M. Magnetically-driven phase transformation strengthening in high entropy alloys. *Nat. Commun.* **9**, 1363 (2018).

5. Togo, A. Phonopy Manual. 1–96 (2013).

6. Chaput, L., Togo, A., Tanaka, I. & Hug, G. Phonon-phonon interactions in transition metals. *Phys. Rev. B - Condens. Matter Mater. Phys.* **84**, 1–6 (2011).

7. Parlinski, K., Li, Z. Q. & Kawazoe, Y. First-principles determination of the soft mode in cubic ZrO2. *Phys. Rev. Lett.* **78**, 4063–4066 (1997).

8. Narasimhan, S. & de Gironcoli, S. Ab initio calculation of the thermal properties of Cu: Performance of the LDA and GGA. *Phys. Rev. B* **65**, 064302 (2002).

9. Zhang, F. X. *et al.* Pressure-induced fcc to hcp phase transition in Ni-based high entropy solid solution alloys. *Appl. Phys. Lett.* **110**, 1–6 (2017).
